# Perceived knowledge, attitudes, and practices toward genital warts and HPV vaccination among college students in Shandong Province, China

**DOI:** 10.3389/fpubh.2026.1931591

**Published:** 2026-10-07

**Authors:** Min Zhu, Guilin Xu, Ningfeng Li, Xinli Tian

**Affiliations:** 1 Department of Obstetrics and Gynecology, Weifang People's Hospital, Weifang, Shandong, China; 2 Department of Infectious Disease Prevention and Control, Huantai Center for Disease Control and Prevention, Zibo, Shandong, China

**Keywords:** attitudes, cross-sectional studies, genital warts, health knowledge, human papillomavirus viruses, papillomavirus vaccines, practice, students

## Abstract

**Objective:**

This study aimed to investigate the perceived knowledge, attitudes, and practices regarding genital warts and HPV vaccination among college students in Shandong Province, China.

**Methods:**

A cross-sectional study was conducted on Chinese college students from March 13 to April 2, 2026, in four universities in Shandong Province, China. Data were collected using a self-administered questionnaire in Chinese that was newly developed for this study and covered demographic characteristics and the three dimensions of the knowledge–attitude–practice (KAP) framework: perceived knowledge (14 items, score range 0–28), attitude (9 items, score range 9–45), and practice (10 items, score range 10–50). Bloom's cut-off criteria were applied to the attainable range of each scale, calculated as (observed score – minimum possible score) / (maximum possible score – minimum possible score) × 100%, and scores were classified as poor (< 60%), moderate (60%−79%), or good (≥80%). Structural equation modeling (SEM) was used to examine the associations among perceived knowledge, attitude, and practice.

**Results:**

A total of 959 participants were included, with 58.39% of participants being male. The mean perceived knowledge, attitude, and practice scores were 15.34 ± 7.64, 35.92 ± 7.42, and 36.84 ± 11.71, corresponding to 54.79%, 74.78%, and 67.10% of the attainable range of each scale, and therefore to poor perceived knowledge and moderate attitude and practice. In the SEM, perceived knowledge was directly associated with attitude (β = 0.119, *P* = 0.012) and with practice (β = 0.461, *P* = 0.014), and attitude was directly associated with practice (β = 0.118, *P* = 0.007). An indirect association between perceived knowledge and practice through attitude was also observed (β = 0.014, *P* = 0.003). Perceived knowledge, attitude, and practice scores were positively correlated with each other (all *P* < 0.001).

**Conclusion:**

College students in Shandong Province, China had poor perceived knowledge and moderate attitudes and practices regarding genital warts and HPV vaccination. Perceived knowledge showed the strongest association with the practice construct in the specified SEM, with attitude acting as a modest indirect association through attitude. Because the design was cross-sectional; these findings represent associations rather than causal effects. Campus-based sexual health education should prioritize accurate HPV-related knowledge, male vaccination awareness, and accessible referral pathways for STI examination and HPV vaccination.

## Introduction

Human papillomavirus (HPV) infection is one of the most common sexually transmitted infections worldwide and is associated with a wide spectrum of diseases, ranging from benign lesions to malignant tumors (1, 2). Persistent infection with high-risk HPV types is closely related to cervical cancer and other anogenital cancers, while low-risk HPV types, especially HPV 6 and 11, are the main causes of genital warts (3). HPV was estimated to cause 620,000 cancer cases in women and 70,000 in men in 2019 (4), and a meta-analysis reported global pooled prevalences of 31% for any genital HPV type and 21% for high-risk HPV among men. In China, a systematic review including more than 850,000 women reported that the prevalence of cervical HPV infection and high-risk HPV infection among women with a normal cervix was 14.3% and 11.3%, respectively (5). Although genital warts are benign, they may cause physical discomfort, psychosocial burden, sexual distress, repeated treatment, and impaired quality of life (6). Current treatments mainly aim to remove visible lesions, but recurrence can occur after treatment (7). Therefore, HPV vaccination is considered an important primary prevention strategy, as it may substantially reduce the burden of HPV-related cancers and genital warts caused by vaccine-covered HPV types, particularly HPV 6 and 11 (8). College students are a key population for HPV prevention, as this period is often accompanied by increasing social independence, sexual health decision-making, and exposure to varied sources of health information, while HPV-related knowledge and vaccination practices remain suboptimal. A study in China reported that actual HPV vaccine uptake among college students was only 3.1%, whereas self-reported willingness to accept HPV vaccination was 36.9% among female students and 24.8% among male students (9). A meta-analysis of 36 studies found that the pooled awareness rate of HPV vaccine among Chinese college students was 40.27%, although awareness did not necessarily translate into actual vaccination uptake (10). Qualitative evidence also showed that many Chinese college students had heard of HPV, but their HPV-related knowledge remained limited and their perceived personal risk of infection was low (11). These findings suggest that college students may have superficial awareness but insufficient accurate knowledge, and that their actual vaccination behavior remains far below their stated willingness.

The knowledge, attitude, and practice (KAP) model provides a structured approach for examining health-related behaviors. It helps assess what individuals know, how they perceive a health issue, and whether their attitudes are translated into preventive actions (12). KAP studies can identify knowledge gaps, misconceptions, behavioral barriers, and targets for health education (13, 14). In HPV-related research, existing studies among college students have mainly focused on HPV vaccine awareness, vaccination intention, vaccine hesitancy, or cervical cancer prevention (15, 16). Studies have shown that HPV vaccine hesitancy among Chinese university students is associated with confidence, complacency, convenience, risk perception, and trust (17, 18). Among male college students, insufficient HPV-related knowledge was associated with weaker intention to recommend HPV vaccination (19). However, fewer studies have examined genital warts and HPV vaccination together within the same KAP framework. This gap is important because genital warts are directly relevant to both sexes and may provide a more immediate and recognizable reason for HPV prevention among college students.

Understanding Shandong college students' KAP toward genital warts and HPV vaccination can help identify misconceptions about HPV transmission, genital warts, vaccine safety and necessity, and male vaccination eligibility, thereby informing targeted interventions in campus health education, clinical counseling, and public health programs. Therefore, this study aimed to investigate the perceived knowledge, attitudes, and practices regarding genital warts and HPV vaccination among college students in Shandong Province, China, and to examine the associations among these three dimensions.

## Methods

### Study design and participants

This cross-sectional study was conducted between March 13 and April 2, 2026, at four universities in Shandong Province, China: Weifang Environmental Engineering Vocational College, Shandong Agricultural University, Shandong Second Medical University, and Shandong Industrial Vocational College. The study population comprised college students enrolled in universities in Shandong Province. The study was approved by the Medical Research Ethics Committee of Weifang People's Hospital (Approval No.: KYLL20260313-10), and written informed consent was obtained from all participants before data collection.

Participants were eligible for inclusion if they met the following criteria: full-time enrollment as college students, age 18 years or older, ability to understand the purpose of the study, and voluntary provision of signed informed consent.

### Questionnaire

The questionnaire used in this study was newly developed for the present survey and was not adapted from an existing validated instrument. An initial item pool was generated on the basis of previous KAP studies (12, 13) and relevant Chinese guidelines and expert consensus documents (20, 21). The draft was then reviewed by a panel of specialists in gynecology, dermatovenereology, and public health, and item wording, item relevance, and content coverage were revised according to their feedback in order to establish content validity. A pilot survey was subsequently conducted among 28 college students to assess the comprehensibility of the items, completion time, and preliminary internal consistency. The overall Cronbach's α coefficient was 0.937, while the coefficients for the perceived knowledge, attitude, and practice sections were 0.973, 0.975, and 0.834, respectively.

Furthermore, construct validity was therefore assessed in the full study sample (*n* = 959). Confirmatory factor analysis of the three-factor measurement model showed acceptable fit, and all standardized factor loadings were statistically significant, ranging from 0.638 to 0.943 (all *P* < 0.001), supporting the intended three-dimensional structure (Supplementary Tables S1, S2). Corrected item-total correlations and Cronbach's α if item deleted were additionally calculated to evaluate item consistency and potential redundancy (Supplementary Table S3).

The final questionnaire was written in Chinese and included four sections: demographic characteristics, perceived knowledge, attitudes, and practices. Demographic information included age, gender, academic year, household registration type, major, HPV vaccination status, and previous exposure to sexual health-related courses or education. The perceived knowledge dimension comprised 14 statements about genital warts and HPV vaccination. For each statement, participants were asked to indicate the extent to which the statement corresponded to their existing understanding. They were instructed to select “very familiar” (2 points) when they had encountered the information and considered that they understood the content clearly, “have heard of it” (1 point) when they had encountered the information but had only partial or incomplete understanding, and “not clear” (0 points) when they were unfamiliar with or unable to understand the information. The total score therefore ranged from 0 to 28 and reflected self-reported familiarity with the content areas covered, rather than objectively verified factual accuracy. The attitude dimension consisted of 9 items assessed using a 5-point Likert agreement scale, scored as 5 for “strongly agree,” 4 for “agree,” 3 for “neutral,” 2 for “disagree,” and 1 for “strongly disagree,” giving a total score range of 9–45. The practice dimension included 10 items covering self-reported preventive behavior and, for some items, behavioral intention and perceived access to services. Each item was rated on a 5-point frequency scale scored from 5 for “always,” 4 for “often,” 3 for “sometimes,” 2 for “occasionally,” to 1 for “never,” giving a total score range of 10–50. The dimension is therefore best understood as a composite index of preventive engagement rather than as a measure of observed behavior alone. Because the attitude and practice scales do not start at zero, Bloom's cut-off criteria were applied to the attainable range of each scale rather than to the maximum possible score. The adjusted percentage was calculated as (observed score – minimum possible score) / (maximum possible score – minimum possible score) × 100%, and each dimension was classified as poor (< 60%), moderate (60%−79%), or good (≥80%) (22, 23).

### Questionnaire distribution and quality control

Data were collected using an online electronic questionnaire. University instructors were contacted to introduce the survey to students during class breaks, explain the inclusion and exclusion criteria, and emphasize that participation was entirely voluntary.

To ensure data quality and improve the reliability of the findings, five predefined screening criteria were applied to identify and exclude invalid questionnaires or responses with potential information bias. First, an attention-check item was included to identify respondents who may not have read the questions carefully. Participants were asked to judge the statement “The Han ethnic group has the smallest population among the 56 ethnic groups in China,” with the response options “true,” “false,” and “not sure.” Because the Han are in fact the most populous of the 56 ethnic groups in China, “false” was the only response regarded as correct, and questionnaires in which “true” or “not sure” was selected were excluded. Second, questionnaires completed in less than 60 s were excluded because adequate time for understanding and responding to the items could not be ensured. Third, responses with obvious numerical outliers, such as an age of 150 years, were removed to reduce data anomalies and potential systematic errors. Fourth, questionnaires showing repetitive response patterns, such as selecting the same option for all items, were excluded as potentially non-genuine responses. Finally, questionnaires containing logical inconsistencies, such as selecting both “never obtained such information” and other sources for the item on main sources of HPV/sexual health information, were also excluded.

### Sample size

The minimum sample size was calculated using the formula for cross-sectional studies: *n* = *Z*^2^ × *p* × (1 – *p*) / *d*^2^ (24). Because no prior estimate was available for the proportion of college students with adequate knowledge, attitudes, and practices regarding genital warts and HPV vaccination in Shandong Province, p was set at 0.50 to obtain the maximum required sample size. With a 95% confidence level, *Z* = 1.96, and an allowable error of 0.05, the minimum required sample size was 384. Considering possible invalid responses, the target sample size was increased. A total of 1,336 questionnaires were collected, and 959 valid questionnaires were finally included, which exceeded the minimum required sample size. Because SEM was a major analytical component of this study, the adequacy of the sample size was additionally considered in relation to model complexity. The specified model contained 33 observed indicators loading on three latent constructs, and the final sample of 959 participants corresponded to approximately 29 participants per observed indicator and exceeded the commonly recommended minimum sample size of 200 cases for SEM (25). Simulation-based guidance further indicates that models with a comparable number of indicators per factor and comparably strong standardized loadings (0.638–0.943 in the present model) achieve adequate power and unbiased estimation at sample sizes well below the one obtained here (26). The achieved sample therefore exceeded both the proportion-based minimum and SEM-specific requirements.

### Statistical analysis

Statistical analysis was conducted using Statistical Package for the Social Sciences 26.0 (IBM, Armonk, NY, USA) and Analysis of Moment Structures 24.0 (IBM, Armonk, NY, USA). Continuous variables were presented as means and standard deviations and assessed for normality. For comparisons between two groups, the independent-samples *t*-test was used for normally distributed continuous variables, while the Wilcoxon–Mann–Whitney test was applied for non-normally distributed variables. For comparisons among three or more groups, one-way analysis of variance was used when the data met the assumptions of normality and homogeneity of variance; otherwise, the Kruskal–Wallis test was employed. Correlations among perceived knowledge, attitude, and practice scores were assessed using Spearman correlation analysis. Structural equation modeling (SEM) was performed on the basis of the knowledge–attitude–practice theoretical framework to examine the associations among perceived knowledge, attitude, and practice, and to evaluate whether attitude mediated the association between perceived knowledge and practice. Direct and indirect effects were estimated and compared within the model. The model was estimated using maximum-likelihood estimation, the default estimator in AMOS. The attitude and practice indicators were five-category Likert-type variables and were treated as continuous, an approach that is generally acceptable when five or more ordered categories are available. The perceived knowledge indicators had only three response categories, and although factor correlations and model fit are estimated adequately by maximum likelihood under these conditions, the corresponding factor loadings may be less accurately recovered than under a categorical estimator; these loadings should therefore be interpreted with appropriate caution (27). Model fit was evaluated using the chi-square to degrees-of-freedom ratio (CMIN/DF), the root mean square error of approximation (RMSEA), the incremental fit index (IFI), the Tucker–Lewis index (TLI), and the comparative fit index (CFI). Acceptable model fit was defined *a priori* as CMIN/DF < 5, RMSEA < 0.08, and IFI, TLI, and CFI > 0.90 (28). Residual covariances were specified between pairs of observed indicators belonging to the same latent dimension. These pairs were identified with reference to modification indices and were retained only where the two items shared closely related content and parallel wording, so that the shared residual variance could reasonably be attributed to overlapping item content rather than to an additional substantive factor. No residual covariance was specified between indicators belonging to different dimensions, and no structural path was added, removed, or reversed on the basis of modification indices; the structural component of the model was fixed *a priori* by the KAP framework. Within-dimension residual covariances of this kind are common in KAP instruments, in which items within a dimension are deliberately worded in parallel to cover adjacent aspects of the same content area, and omitting design-driven residual correlations may itself distort the meaning of the extracted latent variables (29). Because the structural component of the model is saturated at the latent level, with three latent constructs and three directional paths, the confirmatory factor analysis and the full structural model yield identical fit statistics. Total, direct, and indirect effects were estimated using a nonparametric bootstrap procedure with 5,000 resamples; bias-corrected 95% confidence intervals and two-sided bootstrap *P* values were reported, and an effect was considered statistically significant when its confidence interval did not include zero. All comparisons of scores across demographic subgroups were unadjusted, and no multivariable adjustment was performed.

## Results

Initially, this study collected a total of 1,336 questionnaires. The following samples were excluded: (1) 65 participants who did not agree to the use of their data for scientific research; (2) 51 questionnaires completed in less than 60 s; (3) 21 respondents aged under 18 years or with incorrect entries; (4) 20 with logical inconsistencies; (5) 220 who did not select “false” for the attention-check item. No questionnaire met the predefined criterion of a repetitive response pattern, and none was therefore excluded on that ground. Finally, 959 valid questionnaires were included, with a response validity rate of 71.78%.

### Demographic information on participants

This cross-sectional study included 959 college students from Shandong Province, China, with a slight majority of males (58.39%) and most participants were aged 18–20 years. Most students were from rural areas (80.60%) and were in their first academic year (60.79%). Their mean perceived knowledge, attitude, and practice scores were 15.34 ± 7.64 (possible range: 0–28), 35.92 ± 7.42 (possible range: 9–45), and 36.84 ± 11.71 (possible range: 10–50), respectively. Expressed as a proportion of the attainable range of each scale, these values corresponded to 54.79%, 74.78%, and 67.10%, indicating poor perceived knowledge and moderate attitude and practice according to Bloom's cut-off criteria. In unadjusted comparisons, perceived knowledge scores differed significantly by household registration type (*P* = 0.012), major (*P* < 0.001), HPV vaccination status (*P* < 0.001), and receipt of sexual health-related education (*P* < 0.001). Attitude scores differed significantly by gender (*P* < 0.001), major (*P* = 0.002), type of HPV vaccine received (*P* = 0.018), and sexual health education (*P* = 0.015). Practice scores differed significantly by gender (*P* = 0.002), major (*P* < 0.001), type of HPV vaccine received (*P* = 0.009), and sexual health education (*P* < 0.001). The comparison of scores by type of HPV vaccine received was restricted to the 176 participants who had completed or were undergoing HPV vaccination; within this subgroup, 71 (40.34%) had received the bivalent vaccine, 33 (18.75%) the quadrivalent vaccine, and 72 (40.91%) the 9-valent vaccine. All of these comparisons were unadjusted, and no multivariable analysis was performed (Table 1).

**Table 1:** Demographic characteristics of participants and differences in perceived knowledge, attitude, and practice scores.

Variables	*N* (%)	Perceived knowledge, mean ±SD	*P*	Attitude, mean ±SD	*P*	Practice, mean ±SD	*P*
*N* = 959		15.34 ± 7.64		35.92 ± 7.42		36.84 ± 11.71	
**Gender**			0.200		< 0.001		0.002
Male	560 (58.39)	15.06 ± 8.1		35 ± 7.64		35.67 ± 12.28	
Female	399 (41.61)	15.73 ± 6.94		37.2 ± 6.91		38.49 ± 10.66	
**Age**			0.314		0.580		0.408
18	220 (22.94)	15.96 ± 7.93		36.07 ± 7.3		37.52 ± 11.59	
19	351 (36.6)	15.4 ± 7.65		36.28 ± 7.04		37.21 ± 11.68	
20	278 (28.99)	14.86 ± 7.7		35.59 ± 7.81		36.19 ± 12.05	
≥21	110 (11.47)	15.15 ± 6.87		35.26 ± 7.82		35.97 ± 11.22	
**Household registration type**			0.012		0.233		0.102
Urban	186 (19.4)	16.36 ± 7.88		36.41 ± 7.72		37.76 ± 12.37	
Rural	773 (80.6)	15.1 ± 7.57		35.8 ± 7.35		36.62 ± 11.55	
**Current academic year**			0.101		0.121		0.176
First year	583 (60.79)	15.27 ± 7.89		36.14 ± 7.07		37.42 ± 11.49	
Second year	262 (27.32)	14.96 ± 7.14		35.38 ± 7.34		35.69 ± 11.96	
Third year or above	114 (11.89)	16.6 ± 7.41		35.99 ± 9.18		36.55 ± 12.17	
**Major**			< 0.001		0.002		< 0.001
Medical–related major	111 (11.57)	17.73 ± 7.2		37.45 ± 8.23		40.12 ± 11.16	
Non–medical major	848 (88.43)	15.03 ± 7.65		35.71 ± 7.29		36.42 ± 11.72	
**Received the HPV vaccine**			< 0.001		0.171		0.816
Not vaccinated	783 (81.65)	14.84 ± 7.53		35.85 ± 7.23		36.96 ± 11.53	
Fully vaccinated/Currently getting vaccinated	176 (18.35)	17.59 ± 7.77		36.22 ± 8.24		36.31 ± 12.53	
**Type of HPV vaccine received (*****n*** **=** **176)**			0.223		0.018		0.009
Bivalent	71 (40.34)	16.55 ± 7.36		36.08 ± 6.4		33.24 ± 12.56	
Quadrivalent	33 (18.75)	16.91 ± 9.16		33.88 ± 9.46		36.21 ± 12.01	
9–valent	72 (40.91)	18.93 ± 7.38		37.42 ± 9.1		39.39 ± 12.14	
**Sexual health–related education**			< 0.001		0.015		< 0.001
Yes	794 (82.79)	16.39 ± 7.41		36.04 ± 7.59		37.84 ± 11.46	
No	165 (17.21)	10.27 ± 6.66		35.32 ± 6.52		32.05 ± 11.76	

Percentages for the variable “Type of HPV vaccine received” were calculated using the 176 participants who had completed or were undergoing HPV vaccination as the denominator; percentages for all other variables use the total sample (n = 959). All comparisons are unadjusted; no multivariable adjustment was performed.

### Perceived knowledge, attitude, and practice

Responses to the perceived knowledge dimension are shown in Table 2, in which “Very familiar,” “Have heard of it,” and “Not clear” denote clear self-reported understanding, partial understanding, and unfamiliarity, respectively; all percentages were calculated using the total sample (*n* = 959) as the denominator. The three statements with the lowest proportion of participants selecting “Very familiar” were “Genital warts are primarily caused by infection with the HPV.” (K1) with 22.11%, “Genital warts are mainly caused by low-risk HPV types.” (K5) with 22.31%, and “The optimal time for HPV vaccination is before the first sexual activity.” (K12) with 23.46% (Table 2).

**Table 2:** Distribution of perceived knowledge dimension responses.

Knowledge items, *n* (%)	Very familiar	Have heard of it	Not clear
1. Genital warts are primarily caused by infection with the human papillomavirus (HPV).	212 (22.11)	574 (59.85)	173 (18.04)
2. HPV is a virus mainly transmitted through sexual contact.	322 (33.58)	574 (59.85)	63 (6.57)
3. HPV can also be transmitted through indirect contact, such as swimming pools, bathtubs, or toilet seats.	275 (28.68)	565 (58.92)	119 (12.41)
4. HPV infection can occur even in the absence of symptoms.	244 (25.44)	544 (56.73)	171 (17.83)
5. Genital warts are mainly caused by low–risk HPV types (e.g., types 6 and 11).	214 (22.31)	540 (56.31)	205 (21.38)
6. Males can also be infected with HPV and develop related diseases (e.g., genital warts, anal cancer).	232 (24.19)	562 (58.6)	165 (17.21)
7. Genital warts can be controlled with appropriate treatment, but recurrence is possible.	230 (23.98)	572 (59.65)	157 (16.37)
8. HPV vaccination can prevent multiple HPV–related diseases.	258 (26.9)	595 (62.04)	106 (11.05)
9. The HPV vaccines currently approved in China include bivalent, quadrivalent, and 9–valent vaccines.	269 (28.05)	577 (60.17)	113 (11.78)
10. Even if already infected with HPV, vaccination can still protect against other HPV types not yet acquired.	230 (23.98)	573 (59.75)	156 (16.27)
11. HPV vaccines are also effective in preventing HPV–related diseases in males.	228 (23.77)	569 (59.33)	162 (16.89)
12. The optimal time for HPV vaccination is before the first sexual activity.	225 (23.46)	546 (56.93)	188 (19.6)
13. HPV vaccines cannot treat existing HPV infections or genital warts.	228 (23.77)	553 (57.66)	178 (18.56)
14. Women still need to undergo cervical cancer screening after receiving the HPV vaccine.	237 (24.71)	560 (58.39)	162 (16.89)

For each statement, participants indicated the extent to which it corresponded to their existing understanding: “Very familiar” (2 points) indicates that the participant had encountered the information and considered that they understood it clearly; “Have heard of it” (1 point) indicates partial or incomplete understanding; and “Not clear” (0 points) indicates that the participant was unfamiliar with or unable to understand the information. Data are presented as *n* (%), with the total sample (*n* = 959) as the denominator for every item; rows sum to 100%.

Responses to the attitude dimension showed that participants reported positive attitudes across most items. The combined proportion of “agree” and “strongly agree” exceeded 87% for the items assessing the perceived importance of HPV infection (A1, 87.28%), the impact of genital warts (A2, 87.39%), social and emotional stress (A3, 88.63%), the importance of HPV vaccination (A4, 90.93%), male vaccination (A5, 88.64%), vaccine safety (A6, 88.42%), and willingness to receive the vaccine (A7, 87.90%). The corresponding proportions were 86.13% for willingness to recommend vaccination to classmates or friends (A9) and 79.98% for willingness to actively learn more about HPV and vaccination (A8) (Table 3).

**Table 3:** Distribution of attitude dimension responses.

Attitude items, *n* (%)	Strongly disagree	Disagree	Neutral	Agree	Strongly agree
1. I believe HPV infection is a health issue that deserves attention among college students.	73 (7.61)	28 (2.92)	21 (2.19)	477 (49.74)	360 (37.54)
2. I believe genital warts can have a significant impact on an individual's daily life and psychological wellbeing.	55 (5.74)	38 (3.96)	28 (2.92)	542 (56.52)	296 (30.87)
3. I believe HPV infection or genital warts can cause social or emotional stress.	44 (4.59)	38 (3.96)	27 (2.82)	560 (58.39)	290 (30.24)
4. I believe HPV vaccination is very important for preventing related diseases.	37 (3.86)	22 (2.29)	28 (2.92)	543 (56.62)	329 (34.31)
5. I believe males should also receive the HPV vaccine.	35 (3.65)	20 (2.09)	54 (5.63)	542 (56.52)	308 (32.12)
6. I have confidence in the safety of the HPV vaccine.	31 (3.23)	38 (3.96)	42 (4.38)	578 (60.27)	270 (28.15)
7. I am willing to receive the HPV vaccine even if I have not engaged in sexual activity.	28 (2.92)	29 (3.02)	59 (6.15)	539 (56.2)	304 (31.7)
8. I am willing to actively learn more about HPV and vaccination.	26 (2.71)	110 (11.47)	56 (5.84)	454 (47.34)	313 (32.64)
9. I am willing to recommend HPV vaccination to my classmates or friends.	32 (3.34)	22 (2.29)	79 (8.24)	522 (54.43)	304 (31.7)

Each item was rated on a 5–point Likert agreement scale: “Strongly agree” (5 points), “Agree” (4 points), “Neutral” (3 points), “Disagree” (2 points), and “Strongly disagree” (1 point). Data are presented as *n* (%), with the total sample (*n* = 959) as the denominator for every item; rows sum to 100%.

Responses to the practice dimension are shown in Table 4, in which each item was rated on a 5-point frequency scale from “Always” to “Never,” and the cumulative positive rate refers to the combined proportion of “Always” and “Often” responses. Responses to the practice dimension showed that the least positive practices were identified in active information seeking and knowledge of professional examination resources. Specifically, actively seeking information about HPV or sexually transmitted diseases (P3) had the lowest cumulative positive rate (50.99%; 36.08% “Always,” 14.91% “Often”), with 22.42% responding “Occasionally” and 9.38% “Never.” Similarly, knowing where to obtain professional examinations for genital warts or sexually transmitted diseases (P4) showed a low positive rate of 51.62% (34.62% “Always,” 17.00% “Often”), with 21.79% “Occasionally” and 10.32% “Never.” Other practices also demonstrated suboptimal adherence. Participating in school-organized sexual health education activities (P8) had a positive rate of 57.56% (39.83% “Always,” 17.73% “Often”), while encouraging others to learn about prevention (P9) and recommending the HPV vaccine (P10) had rates of 61.11% and 59.64%, respectively. Prompt medical attention when noticing genital or perianal abnormalities (P1) was relatively better (62.99%), yet still leaves room for improvement (Table 4).

**Table 4:** Distribution of practice dimension responses.

Practice items, *n* (%)	Always	Often	Sometimes	Occasionally	Never
1. When I notice abnormal growths, blisters, or unexplained rashes in the genital or perianal area, I seek medical attention promptly.	439 (45.78)	165 (17.21)	106 (11.05)	131 (13.66)	118 (12.3)
2. I actively distinguish between common skin conditions and possible symptoms of sexually transmitted infections (STIs).	397 (41.4)	191 (19.92)	131 (13.66)	157 (16.37)	83 (8.65)
3. I actively seek information about HPV or sexually transmitted diseases.	346 (36.08)	143 (14.91)	165 (17.21)	215 (22.42)	90 (9.38)
4. I know where to obtain professional examinations for genital warts or sexually transmitted diseases.	332 (34.62)	163 (17)	156 (16.27)	209 (21.79)	99 (10.32)
5. During sexual activity, I consistently use condoms to reduce the risk of HPV and genital warts transmission (if applicable).	496 (51.72)	164 (17.1)	97 (10.11)	129 (13.45)	73 (7.61)
6. If I or my sexual partner develop symptoms related to genital warts, I temporarily avoid sexual contact (if applicable).	555 (57.87)	137 (14.29)	84 (8.76)	119 (12.41)	64 (6.67)
7. I have completed or plan to receive the HPV vaccine as soon as possible to prevent genital warts and related diseases.	393 (40.98)	163 (17)	130 (13.56)	165 (17.21)	108 (11.26)
8. I actively participate in sexual health education activities or lectures organized by my school.	382 (39.83)	170 (17.73)	135 (14.08)	203 (21.17)	69 (7.19)
9. I encourage people around me to learn about the prevention of HPV and genital warts.	409 (42.65)	177 (18.46)	139 (14.49)	161 (16.79)	73 (7.61)
10. I actively recommend the HPV vaccine to my friends or family members.	391 (40.77)	181 (18.87)	136 (14.18)	174 (18.14)	77 (8.03)

Each item was rated on a 5–point frequency scale describing how often the participant performed the behavior: “Always” (5 points), “Often” (4 points), “Sometimes” (3 points), “Occasionally” (2 points), and “Never” (1 point). Data are presented as *n* (%), with the total sample (*n* = 959) as the denominator for every item; rows sum to 100%.

### Correlation analysis

In the correlation analysis, positive correlations were found between perceived knowledge and attitude (*r* = 0.184, *P* < 0.001), perceived knowledge and practice (*r* = 0.464, *P* < 0.001), and attitude and practice (*r* = 0.299, *P* < 0.001), respectively (Supplementary Table S4).

### SEM analysis

The SEM showed acceptable fit, and all indices met the prespecified criteria (CMIN/DF = 4.090; RMSEA = 0.057; IFI = 0.960; TLI = 0.954; CFI = 0.960) (Supplementary Table S5); the estimates for the individual paths are presented in Supplementary Table S6 and Figure 1. Perceived knowledge was directly associated with attitude (β = 0.119, 95% CI 0.035–0.182, *P* = 0.012) and with practice (β = 0.461, 95% CI 0.389–0.516, *P* = 0.014), and attitude was directly associated with practice (β = 0.118, 95% CI 0.050–0.187, *P* = 0.007). A significant indirect association between perceived knowledge and practice through attitude was also observed (β = 0.014, 95% CI 0.006–0.034, *P* = 0.003). All confidence intervals were derived from 5,000 bootstrap resamples and excluded zero. Because all three constructs were measured at a single time point, these estimates represent modeled structural associations rather than causal effects (Table 5).

**Figure 1:**
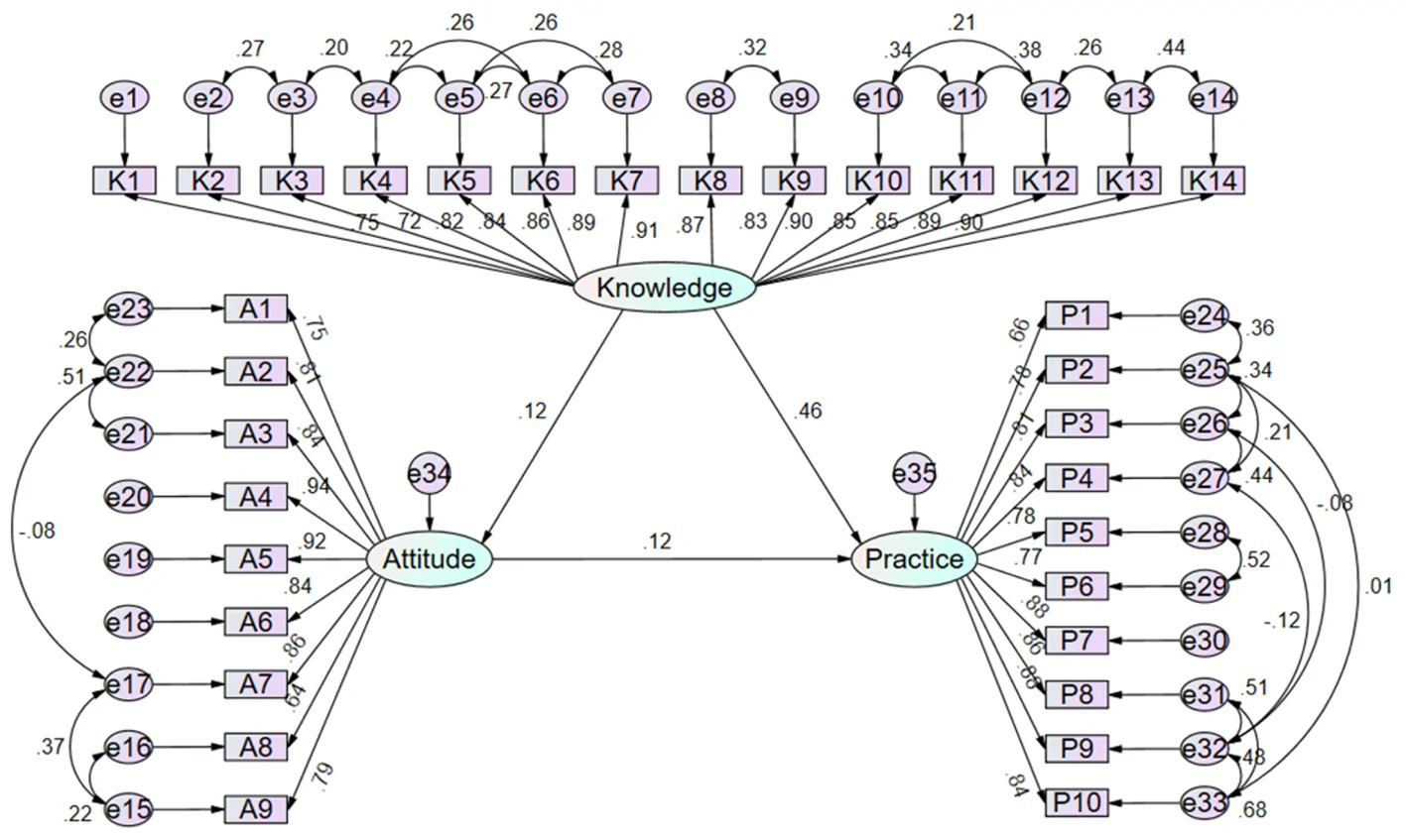
Structural equation model of the relationships among perceived knowledge, attitudes, and practices toward genital warts and HPV vaccination. The model was specified *a priori* on the basis of the knowledge–attitude–practice framework, in which perceived knowledge is hypothesized to be associated with both attitude and practice, and attitude is hypothesized to mediate part of the association between perceived knowledge and practice. Three latent constructs are represented by ellipses and were measured by their respective observed indicators, shown as rectangles: perceived knowledge by items K1–K14, attitude by items A1–A9, and practice by items P1–P10. Single-headed arrows denote standardized regression weights, double-headed arrows denote covariances between residual terms, and e1–e33 denote the residual terms of the observed indicators. The model was estimated by maximum likelihood in AMOS 24.0; total, direct, and indirect effects were tested using 5,000 bootstrap resamples with bias-corrected 95% confidence intervals. Fit indices are given in Supplementary Table S2 and all parameter estimates in Supplementary Table S3.

**Table 5:** SEM analysis of direct and indirect effects.

Model paths	Standardized Total effects		Standardized direct effects		Standardized indirect effects	
	β (95%CI)	*P*	β (95%CI)	*P*	β (95%CI)	*P*
Perceived knowledge → Attitude	0.119 (0.035,0.182)	0.012	0.119 (0.035,0.182)	0.012		
Perceived knowledge → Practice	0.475 (0.409,0.533)	0.009	0.461 (0.389,0.516)	0.014	0.014 (0.006,0.034)	0.003
Attitude → Practice	0.118 (0.050,0.187)	0.007	0.118 (0.050,0.187)	0.007		

β, standardized effect estimate; CI, confidence interval. Effects were estimated by maximum likelihood in AMOS 24.0. Bias–corrected 95% confidence intervals and two-sided P values were obtained from a nonparametric bootstrap procedure with 5,000 resamples; an effect was considered statistically significant when its confidence interval did not include zero. The estimates represent structural associations within the specified cross-sectional model and should not be interpreted as causal effects.

## Discussion

Chinese college students in Shandong Province demonstrated poor perceived knowledge and moderate attitudes and practices regarding genital warts and HPV vaccination. Perceived knowledge showed the strongest association with the practice construct in the specified SEM, and attitude represented a modest indirect association through attitude. Future campus-based and clinical education programs should prioritize accurate HPV-related knowledge, male vaccination awareness, and clear referral pathways for STI assessment and HPV vaccination, while integrating targeted counseling into university health services, dermatology, venereology, and gynecology settings.

The perceived knowledge results indicate that many participants had only partial familiarity with essential HPV-related concepts. Lower levels of understanding were observed for questions related to the etiology of genital warts, the role of low-risk HPV types, the optimal timing of vaccination, and the limitations of HPV vaccines in treating existing infection. Similar patterns have been reported in studies among university students in different regions of China, where awareness of HPV was relatively common, but detailed understanding of transmission, vaccination, and disease prevention remained inadequate (30). This distinction between awareness and accurate knowledge has practical implications because incomplete understanding may reduce the effectiveness of preventive decision-making. Students who are uncertain about vaccine timing or the preventive scope of vaccination may delay vaccination or underestimate their own susceptibility to infection (31). One perceived knowledge item (K3) referred to the possibility of indirect HPV transmission through shared facilities such as swimming pools, bathtubs, or toilet seats. Sexual contact and genital skin-to-skin contact remain the dominant routes of genital HPV transmission, and indirect contact with contaminated surfaces is not an established major route. The item was nevertheless retained after expert discussion because HPV DNA has been detected on the fingers of patients with genital warts and on environmental surfaces in clinical and public settings (32, 33), and because college students spend a substantial part of their daily lives in shared dormitory and public sanitary environments. Beliefs about indirect transmission are consequently a recurrent theme in campus health education and a common source of both anxiety and misconception in this population. The item was therefore intended to capture familiarity with a widely discussed issue and to support advice on personal hygiene in shared environments, rather than to assert that fomite contact is a principal transmission route, and it should be interpreted accordingly.

The correlation and SEM analyses indicate that perceived knowledge was the construct most strongly associated with preventive practice in this sample. Perceived knowledge showed a stronger association with practice than attitude did, while the indirect pathway through attitude was comparatively modest. Because the design was cross-sectional and all constructs were measured simultaneously, these patterns describe structural associations and do not establish temporal order or causal direction. Previous KAP research has also suggested that HPV-related preventive behavior often depends more directly on concrete health knowledge than on general perceptions or approval of vaccination (30). In the present study, higher perceived knowledge scores were associated with higher scores on the overall practice construct. Because the SEM related the latent constructs to one another and did not estimate associations between the perceived knowledge score and each individual practice item, no inference can be drawn from these analyses about specific behaviors such as vaccine intention or symptom-related healthcare seeking. This pattern may reflect the practical nature of HPV prevention. Decisions regarding vaccination, STI examination, and recognition of suspicious symptoms require specific information rather than broad agreement with prevention principles alone.

It should also be noted that, although all three pairwise correlations were statistically significant, their magnitudes differed considerably. The correlation between perceived knowledge and practice was moderate (*r* = 0.464), whereas the correlation between perceived knowledge and attitude was weak (*r* = 0.184) and that between attitude and practice was modest (*r* = 0.299). A plausible explanation is the limited variability of the attitude scores: agreement rates exceeded 86% for eight of the nine attitude items, so the restricted dispersion of this dimension would attenuate any correlation involving it. In addition, favorable attitudes toward HPV prevention in this age group may be shaped largely by general social and media messaging about cervical cancer prevention rather than by detailed understanding of HPV, which would further weaken the association between perceived knowledge and attitude. Comparable dissociations between HPV awareness and vaccine-related attitudes or uptake have been reported among university students in Turkey and the United States (34, 35), suggesting that this pattern is not specific to the Chinese setting.

The unadjusted subgroup comparisons also warrant attention. Medical-related students, vaccinated students, and participants who had received sexual health education had higher perceived knowledge scores; because no multivariable adjustment was performed, these differences should be regarded as unadjusted associations that may be confounded by the interrelationships among these characteristics. Similar associations have been reported in studies examining HPV literacy among healthcare-related student populations (36). Exposure to medical coursework or formal health education may increase opportunities to encounter evidence-based information about HPV infection and vaccination. At the same time, the relatively low knowledge levels observed among non-medical students suggest that current university-based sexual health education may not be reaching the wider student population effectively. Since most participants in this study were non-medical students, reliance on discipline-specific exposure is unlikely to achieve adequate population-level HPV literacy. Universities may therefore need to incorporate HPV-related education into broader student health curricula rather than limiting it to optional lectures or medically oriented programs.

The attitude findings showed generally favorable perceptions toward HPV prevention. Most participants considered HPV infection and genital warts to be important health issues, expressed confidence in HPV vaccination, and showed willingness to actively learn more about HPV and vaccination. This is broadly consistent with previous studies among college students, which reported relatively positive attitudes or willingness toward HPV vaccination despite insufficient detailed knowledge and low vaccine uptake. For example, Deng et al. reported that although HPV vaccine coverage among college students in mainland China was only 3.1%, self-reported vaccine acceptance reached 36.9% among female students and 24.8% among male students (9). Similarly, a cross-sectional study among university students in Qatar found that favorable perceptions toward HPV vaccination did not necessarily translate into actual vaccine uptake, suggesting a persistent gap between attitude and behavior (15). Therefore, the positive attitude observed in the present study should be interpreted as a favorable basis for intervention rather than evidence of adequate preventive practice. Campus-based education should convert this willingness into concrete actions by providing accurate vaccine information, male-specific HPV prevention messages, and clear access pathways for vaccination and STI-related consultation.

The gender differences in attitudes and practices may also reflect the way HPV vaccination has historically been presented in public health communication. Female students demonstrated more positive attitudes and practices than male students, despite the absence of a significant gender difference in perceived knowledge. Related research has suggested that HPV vaccination in China is still frequently associated with cervical cancer prevention, resulting in lower perceived relevance among male populations (37, 38). Although awareness of male vaccination has gradually increased, many male students may continue to regard HPV infection primarily as a women's health issue. We did not conduct gender-stratified analyses of the individual perceived knowledge items, and this interpretation should therefore be regarded as a hypothesis to be tested in future work rather than as a finding of the present dataset. Educational interventions should therefore avoid presenting male vaccination only as a supplementary strategy for protecting female partners. Information provided to students should explicitly address HPV-related disease burden among men, including genital warts and anal cancer, together with the preventive role of vaccination for both sexes (39).

The practice findings revealed an uneven pattern of preventive behavior. Students reported relatively positive responses for condom use, temporary avoidance of sexual contact when symptoms occurred, and willingness to receive HPV vaccination. In contrast, active information seeking and knowledge of professional examination services for genital warts or STIs showed weaker responses. This finding is clinically important because delayed consultation and uncertainty regarding healthcare access may contribute to untreated infection and ongoing transmission. Studies examining STI-related healthcare utilization among young adults have shown that uncertainty about service locations, concerns regarding confidentiality, and limited familiarity with available resources commonly reduce timely healthcare seeking (40). The present findings suggest that these barriers may also affect Chinese university students. Overall practice was classified as moderate rather than good, and this classification should be interpreted cautiously. Several practice items combined actual behavior with behavioral intention or with perceived knowledge of where services can be obtained, and only 176 participants had completed or were undergoing HPV vaccination. The practice score should therefore not be read as evidence of adequate preventive behavior in this population.

An apparently paradoxical pattern was observed in relation to vaccination status. Participants who had completed or were undergoing HPV vaccination had significantly higher perceived knowledge scores than unvaccinated participants (17.59 ± 7.77 versus 14.84 ± 7.53, *P* < 0.001), but their attitude and practice scores did not differ significantly (*P* = 0.171 and *P* = 0.816). Two considerations may help interpret this. First, the attitude items showed very limited variability, with agreement rates above 86% for eight of the nine items, so favorable attitudes were near-universal irrespective of whether a participant had actually been vaccinated, leaving little room for a between-group difference to emerge. Second, the practice dimension is dominated by items concerning information seeking, symptom recognition, service access, and advocacy rather than by vaccination itself, and these behaviors are not necessarily performed more often by students who have already been vaccinated. Taken together, these observations reinforce the interpretation that positive attitudes in this population represent a favorable basis for intervention rather than a marker of preventive engagement, and that vaccination uptake and broader preventive practice appear only loosely coupled.

Participation in school-organized sexual health activities was also less consistent than might be expected given the generally positive attitudes observed in the sample. This may indicate that favorable beliefs alone are insufficient to sustain active engagement with preventive education. The relatively stronger association between perceived knowledge and practice supports this interpretation. Educational activities that rely mainly on general advocacy may have limited impact if they do not provide students with practical information regarding vaccination eligibility, clinical symptoms, referral procedures, and access to STI-related services. Universities could strengthen these programs by integrating HPV-related education into routine student health services and by providing confidential consultation channels linked to campus clinics or local hospitals. The influence of sexual health education on all three KAP dimensions deserves particular emphasis. Participants who had received sexual health-related education showed better perceived knowledge, attitudes, and practices than those without such exposure. Similar associations have been reported in studies evaluating HPV-related education programs among university populations (41). However, the persistence of knowledge gaps despite prior education suggests that existing programs may lack depth or continuity. Brief campaigns or isolated lectures may increase familiarity with HPV terminology without ensuring that students understand vaccine indications, transmission mechanisms, or appropriate healthcare pathways. Educational strategies may therefore need to move beyond awareness promotion toward more applied forms of health communication. Case-based discussions, online modules addressing common misconceptions, and integrated counseling during routine student health visits may provide more effective approaches for improving practical understanding.

The findings also raise broader questions regarding access to HPV-related healthcare resources. Vaccination uptake among participants remained relatively limited, despite positive attitudes toward vaccination. International evidence has shown that HPV vaccine acceptance does not necessarily translate into vaccine uptake when cost, access, or uncertainty regarding vaccination procedures remain unresolved (42). In university settings, students may not know where vaccination services are located, whether appointments are required, or how much vaccination costs. Similar uncertainty may affect STI-related consultation and genital wart examination. Providing clear information regarding available services, confidentiality policies, and referral pathways may therefore be as important as improving knowledge itself.

This study has several limitations. First, although participants were recruited from four universities, all institutions were located in Shandong Province, which may limit the generalizability of the findings to college students in other regions of China with different sociocultural backgrounds, educational resources, and HPV vaccination access. Second, the cross-sectional design precludes causal inference among perceived knowledge, attitudes, and practices, although SEM was used to examine theoretically supported pathways and potential mediating relationships. Third, KAP data were collected through a self-administered questionnaire, which may introduce recall bias and social desirability bias, particularly for sensitive topics related to sexual health; however, anonymous online data collection and predefined quality-control criteria helped reduce invalid or careless responses. Fourth, participants were recruited through classroom-based convenience sampling, which may over-represent students who attend classes regularly and are more engaged with campus activities. Fifth, 220 questionnaires, approximately one-sixth of those initially collected, were excluded because of an incorrect response to the attention-check item; although this procedure was intended to improve data quality, excluded respondents may have differed systematically from those retained, and selection bias cannot be excluded. Sixth, the perceived knowledge dimension measured self-reported familiarity with a set of statements rather than objectively verified factual accuracy, so participants who overestimated their own understanding may have obtained high scores; the very high internal consistency of the perceived knowledge and attitude scales in the pilot survey may also reflect a degree of item redundancy, and item reduction should be considered in future applications of the instrument. Seventh, several practice items combined actual behavior with behavioral intention or with perceived knowledge of where services can be obtained, so the practice score does not constitute a clean measure of observed preventive practice. Eighth, all subgroup comparisons were unadjusted, and the observed differences may be confounded by the interrelationships among gender, major, academic year, vaccination status, and previous sexual health education. Ninth, the perceived knowledge indicators had only three response categories but were treated as continuous under maximum-likelihood estimation. Finally, although the residual covariances included in the model were restricted to indicator pairs within the same dimension that shared overlapping content, and no structural path was modified on the basis of modification indices, the fit statistics are nonetheless partly conditional on this measurement specification and should be interpreted with that in mind. Future multicenter longitudinal studies with broader regional coverage, larger and more diverse samples, and integration of objective vaccination or clinical service-use data are needed to further validate these findings and clarify causal pathways.

Overall, Chinese college students showed poor perceived knowledge and moderate attitudes and practices regarding genital warts and HPV vaccination. Perceived knowledge showed the strongest association with the practice construct in the specified SEM, and attitude represented a modest indirect association through attitude; because the design was cross-sectional, these relationships cannot be interpreted as determinants of preventive practice. Clinically, university health services, dermatology and venereology clinics, gynecology clinics, and vaccination providers should develop targeted education and counseling strategies that strengthen accurate HPV-related knowledge, promote vaccination for both female and male students, and establish accessible referral pathways for STI assessment and HPV vaccination.

## Data Availability

The original contributions presented in the study are included in the article/Supplementary material, further inquiries can be directed to the corresponding author.
