# Supplementary material for: Perceived knowledge, attitudes, and practices toward genital warts and HPV vaccination among college students in Shandong Province, China
Source: Front Public Health. 2026 Oct 7;14:1931591. doi: 10.3389/fpubh.2026.1931591 (PMC13683657; doi:10.3389/fpubh.2026.1931591)
Supplement: Supplementary file 1 [file Table_1.docx]

| Dear Student,  Hello!  We are healthcare professionals from a hospital, and we sincerely invite you to participate in our research study. This study aims to collect information to understand the Knowledge, Attitudes, and Practices (KAP) of Chinese college students regarding genital warts and HPV vaccination, in order to provide evidence for developing scientific intervention strategies. This may help improve outcomes for more people in the future. Your participation is entirely voluntary. This study has been reviewed and approved by the Ethics Committee. If you agree to participate, please read the following information carefully:  1. Please complete the questionnaire based on your actual situation. There are no right or wrong answers. If you have any questions during the process, feel free to ask us. Kindly submit the questionnaire after completion.  2. This study is a simple questionnaire survey and will not cause any physical or psychological harm. However, it may involve some personal information such as gender and age. All information will be kept strictly confidential and will not be disclosed. Please feel assured in completing the questionnaire.  3. As a participant, you have the right to be informed about the study and its progress at any time. If you decide to withdraw, please inform us, and your data will not be included in the final analysis.  Finally, we sincerely thank you for taking the time out of your busy schedule to support our scientific research!  □I have read and understood the above information and agree that the collected data may be used for scientific research.  Informed Consent Signature:  Date of Participation: ______ (year)/ ______(month) / ______(day) | |
| --- | --- |
| **Part 1 Basic Information** | |
| 1. **Gender:** | a. Male b. Female |
| 2. **Age: ______ years (Please set integer validation in the survey system; this note should be removed in the final questionnaire.)** | |
| 3. **Household Registration Type (Hukou):** | a. Urban b. Rural |
| 4. **Current Academic Year:** | a. First year b. Second year c. Third year d. Fourth year e. Fifth year f. Graduate student |
| 5. **Major:** | a. Medical-related major b. Non-medical major |
| 6. **Current Living Arrangement:** | a. On-campus dormitory (shared accommodation) b. Off-campus shared housing/apartment c. Living with family d. Living alone |
| 7. **Have you received the HPV vaccine?** | a. Not vaccinated b. Received 1 dose c. Received 2 doses d. Completed 3 doses/full course |
| 8. **Type of HPV vaccine received:** | a. Bivalent b. Quadrivalent c. 9-valent |
| 9. **Have you received any sexual health-related courses or education?** | a. Yes b. No |
| 10. **Your main sources of HPV/sexual health information (multiple choices allowed):** | a. Doctors/school health education b. Internet/social media c. Classmates/friends d. Others e. Never obtained such information |

**Part 2 Knowledge of Genital Warts and HPV Vaccination**

| 1. **Genital warts are primarily caused by infection with the human papillomavirus (HPV).** | a. very familiar | b. have heard of it | c. not clear |
| --- | --- | --- | --- |
| 2. **HPV is a virus mainly transmitted through sexual contact.** | a. very familiar | b. have heard of it | c. not clear |
| 3. **HPV can also be transmitted through indirect contact, such as swimming pools, bathtubs, or toilet seats.** | a. very familiar | b. have heard of it | c. not clear |
| 4. **HPV infection can occur even in the absence of symptoms.** | a. very familiar | b. have heard of it | c. not clear |
| 5. **Genital warts are mainly caused by low-risk HPV types (e.g., types 6 and 11).** | a. very familiar | b. have heard of it | c. not clear |
| 6. **Males can also be infected with HPV and develop related diseases (e.g., genital warts, anal cancer).** | a. very familiar | b. have heard of it | c. not clear |
| 7. **Genital warts can be controlled with appropriate treatment, but recurrence is possible.** | a. very familiar | b. have heard of it | c. not clear |
| 8. **HPV vaccination can prevent multiple HPV-related diseases.** | a. very familiar | b. have heard of it | c. not clear |
| 9. **The HPV vaccines currently approved in China include bivalent, quadrivalent, and 9-valent vaccines.** | a. very familiar | b. have heard of it | c. not clear |
| 10. **Even if already infected with HPV, vaccination can still protect against other HPV types not yet acquired.** | a. very familiar | b. have heard of it | c. not clear |
| 11. **HPV vaccines are also effective in preventing HPV-related diseases in males.** | a. very familiar | b. have heard of it | c. not clear |
| 12. **The optimal time for HPV vaccination is before the first sexual activity.** | a. very familiar | b. have heard of it | c. not clear |
| 13. **HPV vaccines cannot treat existing HPV infections or genital warts.** | a. very familiar | b. have heard of it | c. not clear |
| 14. **Women still need to undergo cervical cancer screening after receiving the HPV vaccine.** | a. very familiar | b. have heard of it | c. not clear |
| 15. **The Han ethnic group has the smallest population among the 56 ethnic groups in China.** | a. true | b. false | c. not clear |

| **Part 3 Attitudes Toward Genital Warts and HPV Vaccination** | | | | | |
| --- | --- | --- | --- | --- | --- |
| 1. **I believe HPV infection is a health issue that deserves attention among college students.** | a. strong disagree | b. disagree | c. neutral | d. agree | e. strongly agree |
| 2. **I believe genital warts can have a significant impact on an individual’s daily life and psychological well-being.** | a. strong disagree | b. disagree | c. neutral | d. agree | e. strongly agree |
| 3. **I believe HPV infection or genital warts can cause social or emotional stress.** | a. strong disagree | b. disagree | c. neutral | d. agree | e. strongly agree |
| 4. **I believe HPV vaccination is very important for preventing related diseases.** | a. strong disagree | b. disagree | c. neutral | d. agree | e. strongly agree |
| 5. **I believe males should also receive the HPV vaccine.** | a. strong disagree | b. disagree | c. neutral | d. agree | e. strongly agree |
| 6. **I have confidence in the safety of the HPV vaccine.** | a. strong disagree | b. disagree | c. neutral | d. agree | e. strongly agree |
| 7. **I am willing to receive the HPV vaccine even if I have not engaged in sexual activity.** | a. strong disagree | b. disagree | c. neutral | d. agree | e. strongly agree |
| 8. **I am willing to actively learn more about HPV and vaccination.** | a. strong disagree | b. disagree | c. neutral | d. agree | e. strongly agree |
| 9. **I am willing to recommend HPV vaccination to my classmates or friends.** | a. strong disagree | b. disagree | c. neutral | d. agree | e. strongly agree |

| **Part 4 Practices Related to Genital Warts and HPV Vaccination** | | | | | |
| --- | --- | --- | --- | --- | --- |
| 1. **When I notice abnormal growths, blisters, or unexplained rashes in the genital or perianal area, I seek medical attention promptly.** | a. always | b. often | c. sometimes | d. occasionally | e. never |
| 2. **I actively distinguish between common skin conditions and possible symptoms of sexually transmitted infections (STIs).** | a. always | b. often | c. sometimes | d. occasionally | e. never |
| 3. **I actively seek information about HPV or sexually transmitted diseases.** | a. always | b. often | c. sometimes | d. occasionally | e. never |
| 4. **I know where to obtain professional examinations for genital warts or sexually transmitted diseases.** | a. always | b. often | c. sometimes | d. occasionally | e. never |
| 5. **During sexual activity, I consistently use condoms to reduce the risk of HPV and genital warts transmission (if applicable).** | a. always | b. often | c. sometimes | d. occasionally | e. never |
| 6. **If I or my sexual partner develop symptoms related to genital warts, I temporarily avoid sexual contact (if applicable).** | a. always | b. often | c. sometimes | d. occasionally | e. never |
| 7. **I have completed or plan to receive the HPV vaccine as soon as possible to prevent genital warts and related diseases.** | a. always | b. often | c. sometimes | d. occasionally | e. never |
| 8. **I actively participate in sexual health education activities or lectures organized by my school.** | a. always | b. often | c. sometimes | d. occasionally | e. never |
| 9. **I encourage people around me to learn about the prevention of HPV and genital warts.** | a. always | b. often | c. sometimes | d. occasionally | e. never |
| 10. **I actively recommend the HPV vaccine to my friends or family members.** | a. always | b. often | c. sometimes | d. occasionally | e. never |
