# Supplementary material for: Perceived knowledge, attitudes, and practices toward genital warts and HPV vaccination among college students in Shandong Province, China
Source: Front Public Health. 2026 Oct 7;14:1931591. doi: 10.3389/fpubh.2026.1931591 (PMC13683657; doi:10.3389/fpubh.2026.1931591)
Supplement: Supplementary file 2 [file Table_2.docx]

**Supplementary table 1. Confirmatory factor analysis fit indicators**

| **Model fit indicator** | **Ref.** | **Measured results** |
| --- | --- | --- |
| **CMIN/DF** | 1-3 excellent，3-5 good | 4.090 |
| **RMSEA** | <0.08 good | 0.057 |
| **IFI** | >0.90 good | 0.960 |
| **TLI** | >0.90 good | 0.954 |
| **CFI** | >0.90 good | 0.960 |

**Supplementary table 2. Standardized factor loadings from confirmatory factor analysis of the perceived knowledge, attitude, and practice constructs**

|  |  |  | **Estimate** | **Standardized Estimate** | **S.E.** | **C.R.** | **P** |
| --- | --- | --- | --- | --- | --- | --- | --- |
| K1 | <--- | Perceived knowledge | 1.000 | 0.753 |  |  |  |
| K2 | <--- | Perceived knowledge | 0.861 | 0.715 | 0.037 | 23.138 | <0.001 |
| K3 | <--- | Perceived knowledge | 1.061 | 0.817 | 0.039 | 26.982 | <0.001 |
| K4 | <--- | Perceived knowledge | 1.158 | 0.845 | 0.041 | 28.059 | <0.001 |
| K5 | <--- | Perceived knowledge | 1.197 | 0.864 | 0.042 | 28.787 | <0.001 |
| K6 | <--- | Perceived knowledge | 1.194 | 0.891 | 0.040 | 29.892 | <0.001 |
| K7 | <--- | Perceived knowledge | 1.202 | 0.907 | 0.039 | 30.581 | <0.001 |
| K8 | <--- | Perceived knowledge | 1.087 | 0.869 | 0.037 | 29.041 | <0.001 |
| K9 | <--- | Perceived knowledge | 1.068 | 0.834 | 0.039 | 27.620 | <0.001 |
| K10 | <--- | Perceived knowledge | 1.191 | 0.900 | 0.039 | 30.317 | <0.001 |
| K11 | <--- | Perceived knowledge | 1.138 | 0.854 | 0.040 | 28.430 | <0.001 |
| K12 | <--- | Perceived knowledge | 1.170 | 0.855 | 0.041 | 28.450 | <0.001 |
| K13 | <--- | Perceived knowledge | 1.202 | 0.887 | 0.040 | 29.772 | <0.001 |
| K14 | <--- | Perceived knowledge | 1.209 | 0.899 | 0.040 | 30.250 | <0.001 |
| A9 | <--- | Attitude | 1.000 | 0.786 |  |  |  |
| A8 | <--- | Attitude | 1.286 | 0.638 | 0.055 | 23.500 | <0.001 |
| A7 | <--- | Attitude | 1.081 | 0.863 | 0.028 | 38.265 | <0.001 |
| A6 | <--- | Attitude | 1.068 | 0.841 | 0.036 | 29.696 | <0.001 |
| A5 | <--- | Attitude | 1.174 | 0.922 | 0.035 | 33.703 | <0.001 |
| A4 | <--- | Attitude | 1.211 | 0.943 | 0.035 | 34.804 | <0.001 |
| A3 | <--- | Attitude | 1.152 | 0.840 | 0.039 | 29.624 | <0.001 |
| A2 | <--- | Attitude | 1.168 | 0.813 | 0.041 | 28.329 | <0.001 |
| A1 | <--- | Attitude | 1.181 | 0.747 | 0.046 | 25.447 | <0.001 |
| P1 | <--- | Practice | 1.000 | 0.665 |  |  |  |
| P2 | <--- | Practice | 1.096 | 0.781 | 0.041 | 26.523 | <0.001 |
| P3 | <--- | Practice | 1.175 | 0.811 | 0.053 | 22.169 | <0.001 |
| P4 | <--- | Practice | 1.217 | 0.838 | 0.053 | 22.793 | <0.001 |
| P5 | <--- | Practice | 1.084 | 0.777 | 0.050 | 21.479 | <0.001 |
| P6 | <--- | Practice | 1.057 | 0.773 | 0.049 | 21.383 | <0.001 |
| P7 | <--- | Practice | 1.309 | 0.882 | 0.055 | 23.837 | <0.001 |
| P8 | <--- | Practice | 1.210 | 0.855 | 0.052 | 23.179 | <0.001 |
| P9 | <--- | Practice | 1.226 | 0.877 | 0.052 | 23.609 | <0.001 |
| P10 | <--- | Practice | 1.187 | 0.840 | 0.052 | 22.819 | <0.001 |

**Supplementary Table S3. Corrected item-total correlations and Cronbach’s α if item deleted for questionnaire items**

| **Item** | **Corrected item-total correlation** | **Cronbach’s α if item deleted** |
| --- | --- | --- |
| K1 | 0.744 | 0.974 |
| K2 | 0.713 | 0.974 |
| K3 | 0.814 | 0.973 |
| K4 | 0.847 | 0.972 |
| K5 | 0.863 | 0.972 |
| K6 | 0.889 | 0.971 |
| K7 | 0.9 | 0.971 |
| K8 | 0.855 | 0.972 |
| K9 | 0.818 | 0.973 |
| K10 | 0.889 | 0.971 |
| K11 | 0.851 | 0.972 |
| K12 | 0.844 | 0.972 |
| K13 | 0.874 | 0.972 |
| K14 | 0.887 | 0.971 |
| A1 | 0.737 | 0.939 |
| A2 | 0.793 | 0.935 |
| A3 | 0.824 | 0.933 |
| A4 | 0.903 | 0.93 |
| A5 | 0.873 | 0.931 |
| A6 | 0.806 | 0.935 |
| A7 | 0.843 | 0.933 |
| A8 | 0.631 | 0.952 |
| A9 | 0.786 | 0.936 |
| P1 | 0.673 | 0.956 |
| P2 | 0.801 | 0.95 |
| P3 | 0.813 | 0.949 |
| P4 | 0.819 | 0.949 |
| P5 | 0.783 | 0.951 |
| P6 | 0.776 | 0.951 |
| P7 | 0.837 | 0.948 |
| P8 | 0.845 | 0.948 |
| P9 | 0.86 | 0.947 |
| P10 | 0.829 | 0.949 |

**Supplementary table 4. Correlation analysis among Perceived knowledge, attitude, and practice scores**

|  | Perceived knowledge | Attitude | Practice |
| --- | --- | --- | --- |
| Perceived knowledge | 1 |  |  |
| Attitude | 0.184 (P<0.001) | 1 |  |
| Practice | 0.464 (P<0.001) | 0.299 (P<0.001) | 1 |

**Supplementary table 5. SEM fit indicators**

| **Model fit indicator** | **Ref.** | **Measured results** |
| --- | --- | --- |
| **CMIN/DF** | 1-3 excellent，3-5 good | 4.090 |
| **RMSEA** | <0.08 good | 0.057 |
| **IFI** | >0.90 good | 0.960 |
| **TLI** | >0.90 good | 0.954 |
| **CFI** | >0.90 good | 0.960 |

**Supplementary table 6. Estimated total effect coefficient**

|  |  |  | **Estimate** | **Standardized Estimate** | **S.E.** | **C.R.** | **P** |
| --- | --- | --- | --- | --- | --- | --- | --- |
| Attitude | <--- | Perceived knowledge | 0.173 | 0.119 | 0.049 | 3.541 | <0.001 |
| Practice | <--- | Attitude | 0.166 | 0.118 | 0.043 | 3.823 | <0.001 |
| Practice | <--- | Perceived knowledge | 0.941 | 0.461 | 0.076 | 12.429 | <0.001 |
| K1 | <--- | Perceived knowledge | 1.000 | 0.753 |  |  |  |
| K2 | <--- | Perceived knowledge | 0.861 | 0.715 | 0.037 | 23.138 | <0.001 |
| K3 | <--- | Perceived knowledge | 1.061 | 0.817 | 0.039 | 26.982 | <0.001 |
| K4 | <--- | Perceived knowledge | 1.158 | 0.845 | 0.041 | 28.059 | <0.001 |
| K5 | <--- | Perceived knowledge | 1.197 | 0.864 | 0.042 | 28.787 | <0.001 |
| K6 | <--- | Perceived knowledge | 1.194 | 0.891 | 0.040 | 29.892 | <0.001 |
| K7 | <--- | Perceived knowledge | 1.202 | 0.907 | 0.039 | 30.581 | <0.001 |
| K8 | <--- | Perceived knowledge | 1.087 | 0.869 | 0.037 | 29.041 | <0.001 |
| K9 | <--- | Perceived knowledge | 1.068 | 0.834 | 0.039 | 27.620 | <0.001 |
| K10 | <--- | Perceived knowledge | 1.191 | 0.900 | 0.039 | 30.317 | <0.001 |
| K11 | <--- | Perceived knowledge | 1.138 | 0.854 | 0.040 | 28.430 | <0.001 |
| K12 | <--- | Perceived knowledge | 1.170 | 0.855 | 0.041 | 28.450 | <0.001 |
| K13 | <--- | Perceived knowledge | 1.202 | 0.887 | 0.040 | 29.772 | <0.001 |
| K14 | <--- | Perceived knowledge | 1.209 | 0.899 | 0.040 | 30.250 | <0.001 |
| A9 | <--- | Attitude | 1.000 | 0.786 |  |  |  |
| A8 | <--- | Attitude | 1.286 | 0.638 | 0.055 | 23.500 | <0.001 |
| A7 | <--- | Attitude | 1.081 | 0.863 | 0.028 | 38.265 | <0.001 |
| A6 | <--- | Attitude | 1.068 | 0.841 | 0.036 | 29.696 | <0.001 |
| A5 | <--- | Attitude | 1.174 | 0.922 | 0.035 | 33.703 | <0.001 |
| A4 | <--- | Attitude | 1.211 | 0.943 | 0.035 | 34.804 | <0.001 |
| A3 | <--- | Attitude | 1.152 | 0.840 | 0.039 | 29.624 | <0.001 |
| A2 | <--- | Attitude | 1.168 | 0.813 | 0.041 | 28.329 | <0.001 |
| A1 | <--- | Attitude | 1.181 | 0.747 | 0.046 | 25.447 | <0.001 |
| P1 | <--- | Practice | 1.000 | 0.665 |  |  |  |
| P2 | <--- | Practice | 1.096 | 0.781 | 0.041 | 26.523 | <0.001 |
| P3 | <--- | Practice | 1.175 | 0.811 | 0.053 | 22.169 | <0.001 |
| P4 | <--- | Practice | 1.217 | 0.838 | 0.053 | 22.793 | <0.001 |
| P5 | <--- | Practice | 1.084 | 0.777 | 0.050 | 21.479 | <0.001 |
| P6 | <--- | Practice | 1.057 | 0.773 | 0.049 | 21.383 | <0.001 |
| P7 | <--- | Practice | 1.309 | 0.882 | 0.055 | 23.837 | <0.001 |
| P8 | <--- | Practice | 1.210 | 0.855 | 0.052 | 23.179 | <0.001 |
| P9 | <--- | Practice | 1.226 | 0.877 | 0.052 | 23.609 | <0.001 |
| P10 | <--- | Practice | 1.187 | 0.840 | 0.052 | 22.819 | <0.001 |
